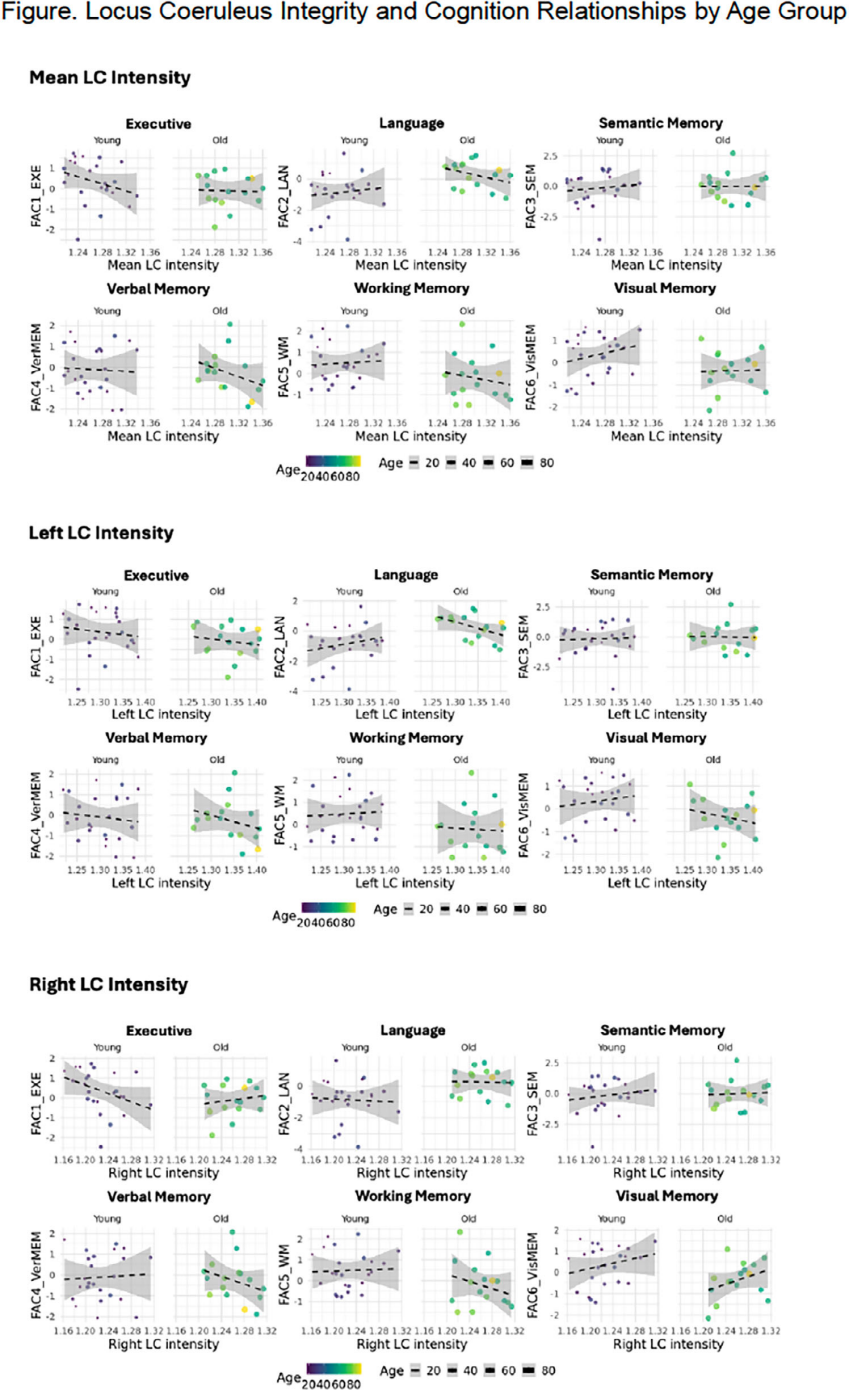# Age‐related differences in Locus Coeruleus integrity and their differential relationships with cognition

**DOI:** 10.1002/alz70856_106290

**Published:** 2026-01-07

**Authors:** Ji‐Hyun Kim, Xiaoyang Hu, Jasmine Ranieri, Heidi I.L. Jacobs, Hwamee Oh

**Affiliations:** ^1^ Brown University, Providence, RI, USA; ^2^ Athinoula A. Martinos Center for Biomedical Imaging, Massachusetts General Hospital, Harvard Medical School, Boston, MA, USA

## Abstract

**Background:**

The locus coeruleus (LC), a key source of norepinephrine production, plays an essential role in arousal and cognitive functions. Abnormal tau begins to appear in the brain stem, the LC in particular, even in young adulthood long before brain tau and amyloid pathology. To what extent LC integrity affects cognitive performance accounting for age is unknown. In this study, we examined age‐related differences in LC integrity and its relationship with cognition in the lifespan sample.

**Methods:**

A total of 124 cognitively normal adults (72 older (mean age: 68.85.9; 52 females) and 52 young (mean age: 24.25.4; 35 females)) from Brown Multimodal Imaging of Cognition, Aging, and Alzheimer's Disease (MICAAD) study underwent extensive neuropsychological assessment and a subset of 40 adults completed LC MT‐MRI imaging. Principal component (PC) analyses were applied to a set of neuropsychological tests that included standard measures of verbal and visual memory, executive function, language, semantic memory, and working memory. LC intensity was calculated by normalizing the LC signal relative to the pontine tegmentum and five highest intensity voxels from each hemisphere were averaged to compute the left LC, right LC, and mean LC (left LC and right LC combined) intensity values. Multiple regressions were performed to assess the relationships between cognitive score and LC intensity, age group, and their interaction, with sex as a covariate.

**Results:**

Across the whole sample, age group‐related differences were found in executive function, language, and visual memory. LC intensity was significantly higher in older compared to young adults and in the left compared to the right hemisphere across both age groups. No direct association between mean LC intensity and cognitive domains was found in either age group, while significant relationships were found in the left LC intensity and language and the right LC intensity and working memory only among young adults.

**Conclusions:**

While there is an age‐related difference in LC intensity, the relationships between LC intensity and cognition are domain‐specific and age‐group dependent. Further research is warranted to evaluate the contribution of laterality and resilience factors in the LC integrity and cognition relationship in a larger sample.